# Dynamic Inflammatory and Erythrocyte-Derived Biomarkers in Newly Diagnosed Multiple Myeloma: A Longitudinal Analysis of Classical and Novel Indices

**DOI:** 10.3390/biomedicines14081839

**Published:** 2026-08-15

**Authors:** Alexandra-Ştefania Stroe-Ionescu, Lidia Boldeanu, Ana Maria Pǎtraşcu, Janina-Georgiana Goanțǎ, Isabela Siloși, Mohamed-Zakaria Assani, Ionela Rotaru, Alina Daniela Tǎnase, Mihail Virgil Boldeanu

**Affiliations:** 1 Doctoral School, University of Medicine and Pharmacy of Craiova, 200349 Craiova, Romania; alexandra.stroe@umfcv.ro (A.-Ş.S.-I.); mohamed.assani@umfcv.ro (M.-Z.A.); 2 Department of Microbiology, Faculty of Medicine, University of Medicine and Pharmacy of Craiova, 200349 Craiova, Romania; lidia.boldeanu@umfcv.ro; 3 Department of Hematology, Faculty of Medicine, University of Medicine and Pharmacy of Craiova, 200349 Craiova, Romania; anamaria.patrascu@umfcv.ro (A.M.P.); janina.goanta@umfcv.ro (J.-G.G.); 4 Department of Immunology, Faculty of Medicine, University of Medicine and Pharmacy of Craiova, 200349 Craiova, Romania; isabela.silosi@umfcv.ro (I.S.); mihail.boldeanu@umfcv.ro (M.V.B.); 5 Department of Hematology and Bone Marrow Transplantation, Fundeni Clinical Institute, 022328 Bucharest, Romania

**Keywords:** multiple myeloma, inflammatory biomarkers, mean corpuscular volume-to-lymphocyte ratio (MCVL), cumulative inflammatory index (IIC), induction therapy, progression-free survival, biomarker dynamics, prognostic markers

## Abstract

**Background/Objectives:** Inflammatory and hematologic indices derived from routine blood tests have been increasingly investigated as prognostic biomarkers in multiple myeloma (MM). However, their clinical utility remains inconsistent, and data on novel composite indices, such as the mean corpuscular volume-to-lymphocyte ratio (MCVL) and the cumulative inflammatory index (IIC), are lacking in MM. **Methods:** We conducted a retrospective study including 122 patients with newly diagnosed MM. Hematologic and inflammatory indices were evaluated at baseline and after four cycles of induction therapy. Associations with progression-free survival (PFS) and overall survival (OS) were assessed using Kaplan–Meier analysis, Cox regression models, and receiver operating characteristic (ROC) curve analysis. **Results:** Baseline inflammatory biomarkers, including neutrophil-to-lymphocyte ratio (NLR), platelet-to-lymphocyte ratio (PLR), monocyte-to-lymphocyte ratio (MLR), systemic immune–inflammation index (SII), MCVL, and IIC, were not significantly associated with PFS or OS. ROC analysis demonstrated poor discriminative ability for all evaluated markers at both baseline and post-induction timepoints (AUC values close to or below 0.50). In contrast, post-induction inflammatory indices, particularly PLR, MLR, AISI, and SIRI, were significantly associated with PFS in both univariable and multivariable Cox regression analyses. Neither baseline nor post-induction MCVL and IIC showed independent prognostic value. **Conclusions:** Baseline inflammatory and erythrocyte-derived indices, including the novel composite markers MCVL and IIC, have limited prognostic utility in MM. In contrast, dynamic changes in inflammatory biomarkers during treatment may provide more clinically relevant information regarding disease progression. These findings support integrating longitudinal biomarker assessment into future risk stratification models for MM.

## 1. Introduction

Multiple myeloma (MM) is a complex hematologic malignancy characterized by clonal plasma-cell proliferation and end-organ damage, including hypercalcemia, renal impairment, anemia, and osteolytic bone disease [1]. Recent epidemiological data indicate a rising global burden, with approximately 188,000 new cases reported in 2022 and a projected increase in incidence of up to 71% by 2045 [2]. Although MM remains incurable, therapeutic advances involving immunomodulatory drugs, proteasome inhibitors, and anti-CD38 monoclonal antibodies have significantly improved survival and quality of life [3,4]. Inflammation plays a central role in MM pathogenesis, as interactions between malignant plasma cells, bone marrow stromal cells, and immune-cell populations promote cytokine secretion, tumor growth, and immune dysregulation [5,6,7].

In this context, systemic inflammatory markers derived from routine complete blood count parameters have emerged as accessible and cost-effective prognostic tools. Ratios such as the neutrophil-to-lymphocyte ratio (NLR), platelet-to-lymphocyte ratio (PLR), and monocyte-to-lymphocyte ratio (MLR), together with composite indices including the systemic immune–inflammation index (SII), aggregate index of systemic inflammation (AISI), and systemic inflammation response index (SIRI), integrate different components of the host inflammatory and immune response. Elevated values of these indices have been associated with greater tumor burden, aggressive disease characteristics, and inferior outcomes in solid tumors and hematologic malignancies, including MM [8,9,10,11,12,13].

Erythrocyte-related parameters may provide additional information regarding systemic inflammation and bone marrow dysfunction. Composite indices such as the mean corpuscular volume-to-lymphocyte ratio (MCVL) and the cumulative inflammatory index (IIC), calculated as (MCV × RDW × neutrophil count)/(lymphocyte count × 1000), have been investigated in malignant and inflammatory disorders [14,15,16,17]. However, their prognostic relevance in MM remains unknown. Red cell distribution width (RDW), a marker of erythrocyte-size heterogeneity, has been associated with inflammation, nutritional deficiencies, disease severity, and reduced survival in MM [18,19,20,21].

While most studies have focused on baseline inflammatory markers, induction therapy induces substantial changes in tumor burden and immune dynamics. Therefore, evaluating the longitudinal evolution of these indices may provide additional prognostic insight. Assessing variations in NLR, PLR, MLR, RDW, and SII, as well as novel indices such as MCVL and IIC, from diagnosis to post-induction may improve early prediction of treatment response and long-term outcomes.

This study aims to investigate inflammatory and hematologic indices in patients with newly diagnosed MM at baseline and after four cycles of induction therapy, and to evaluate their associations with progression-free survival (PFS) and overall survival (OS). By integrating established and emerging composite biomarkers into a longitudinal framework, we aim to assess their utility as early, cost-effective prognostic stratification tools in routine MM management.

## 2. Materials and Methods

### 2.1. Study Design and Patient Selection

This retrospective single-center observational study included 122 patients with newly diagnosed MM treated at the Hematology Department of the Clinical Municipal Hospital Filantropia Craiova, Romania, between January 2018 and October 2024. The diagnosis of MM was established according to the International Myeloma Working Group (IMWG) criteria applicable at the time of diagnosis [22].

The study was conducted in accordance with the Declaration of Helsinki and was approved by the Ethics Committee of the Clinical Municipal Hospital Filantropia (no. 5025/4 March 2025), Dolj, Romania, and by the Ethics Committee of the University of Medicine and Pharmacy of Craiova (no. 151/4 April 2025), Dolj, Romania.

Eligible patients were required to have clinical and laboratory data available at diagnosis, including complete blood count (CBC)-derived inflammatory indices, and to undergo a repeat hematologic evaluation after four cycles of induction therapy. Only patients with available follow-up data for relapse/progression and survival assessment were included in the final analysis.

Patients were excluded if they had: (1) active infection at the time of blood sampling; (2) concomitant autoimmune or chronic inflammatory disease likely to influence hematologic inflammatory indices; (3) another active malignancy; (4) missing key laboratory or follow-up data; or (5) prior anti-myeloma treatment before baseline assessment.

Demographic, clinical, laboratory, and outcome data were extracted from the institutional electronic medical records and local hematology database. The collected variables included age, sex, MM subtype, light-chain type, disease stage, plasma cell marrow infiltration, inflammatory parameters, biochemical variables, transplant status, induction regimen, date of diagnosis, date of relapse/progression, date of death, and survival status at last follow-up.

### 2.2. Clinical and Laboratory Assessment

Baseline assessment was defined as the time of diagnosis, before initiation of induction therapy. Post-treatment assessment was performed after completion of four cycles of induction therapy, using CBC and biochemical parameters recorded at the corresponding re-evaluation time point.

The following laboratory variables were collected at baseline:

A CBC was performed using peripheral venous blood collected in Vacutainer tubes containing ethylene-diamine-tetra-acetic acid (EDTA) as an anticoagulant. Utilizing flow cytometry and Coulter’s principle, we obtained an extended leukocyte formula of 5 diff (Alinity, Abbott Laboratories, Abbott Park, IL, USA) and determined the hematologic markers: hemoglobin (Hb), white blood cells (WBCs), neutrophils (NEUs), lymphocytes (LYMs), monocytes (MONs), platelets (PLTs), and hematocrit (Ht). The following indices were analyzed:

– neutrophil-to-lymphocyte ratio (NLR) = absolute neutrophil count/absolute lymphocyte count;
– platelet-to-lymphocyte ratio (PLR) = platelet count/absolute lymphocyte count;
– monocyte-to-lymphocyte ratio (MLR) = absolute monocyte count/absolute lymphocyte count;
– systemic immune–inflammation index (SII) = platelet count × absolute neutrophil count/absolute lymphocyte count;
– aggregate index of systemic inflammation (AISI) = (neutrophils × monocytes × platelets)/lymphocytes;
– systemic inflammation response index (SIRI) = (neutrophils × monocytes)/lymphocytes;
– red cell distribution width (RDW), expressed as a percentage (%), as provided by the hematology analyzer;
– mean corpuscular volume-to-lymphocyte ratio (MCVL) = mean corpuscular volume (MCV)/absolute lymphocyte count;
– cumulative inflammatory index (IIC) = (mean corpuscular volume × red cell distribution width × absolute neutrophil count)/(absolute lymphocyte count × 1000);
– erythrocyte sedimentation rate (ESR), C-reactive protein (CRP), lactate dehydrogenase (LDH), creatinine, and calcium.

At the post-induction timepoint, repeat values were available for the main hematologic and inflammatory parameters, including Hb, WBCs, NEUs, LYMs, MONs, PLTs, Ht, CRP, ESR, LDH, RDW, MCV, and all CBC-derived indices.

Clinical variables included MM isotype, κ/λ light-chain restriction, disease stage, and autologous stem cell transplantation status.

To assess treatment-related variation, dynamic changes in each biomarker were defined as the difference between post-induction and baseline values (Δindex=post−induction value−baseline value).

### 2.3. Study Endpoints

The primary endpoints of the study were PFS and OS. For the purposes of this analysis, PFS was defined as the interval from diagnosis to the first documented relapse/progression recorded in the database. Patients without a recorded event were censored at the date of last follow-up. OS was defined as the interval from the date of diagnosis to death from any cause. Patients who were alive at the last follow-up were censored on that date.

Secondary objectives included evaluating changes in inflammatory indices between diagnosis and post-induction assessment and assessing the associations of baseline, post-induction, and dynamic biomarker values with survival outcomes.

### 2.4. Statistical Analysis

Statistical analysis was performed using GraphPad Prism 11.0.1 (90) (GraphPad Software, San Diego, CA, USA). Continuous variables were tested for normality using the Shapiro–Wilk test. Normally distributed variables were expressed as mean ± standard deviation (SD), whereas non-normally distributed variables were reported as median and interquartile range (IQR). Categorical variables were presented as frequencies and percentages.

Comparisons between independent groups were performed using Student’s *t*-test for normally distributed continuous variables and the Mann–Whitney U test for non-normally distributed variables. Categorical variables were compared using the chi-square test or Fisher’s exact test, as appropriate. Paired comparisons between baseline and post-induction values were performed using the paired *t*-test or the Wilcoxon signed-rank test, depending on variable distribution.

Survival curves for PFS and OS were estimated using the Kaplan–Meier method and compared using the log-rank test. The prognostic impact of baseline, post-induction, and dynamic inflammatory indices was evaluated using Cox proportional hazards regression. Multivariable Cox regression models were adjusted for age, disease stage, transplant status, serum creatinine, lactate dehydrogenase, and bone marrow plasma cell percentage. Hazard ratios (HRs) and 95% confidence intervals (CIs) were reported. The proportional hazards assumption was formally evaluated using Schoenfeld residual-based tests for each Cox regression model.

To avoid model overfitting and instability due to mathematical overlap among CBC-derived indices, strongly correlated inflammatory markers were not included simultaneously in the same multivariable model. Because several CBC-derived biomarkers are mathematically interrelated, the novel erythrocyte-based indices MCVL and IIC were evaluated in dedicated multivariable models in order to better assess their independent prognostic contribution beyond conventional inflammatory markers and standard clinical variables.

Receiver operating characteristic (ROC) curve analysis was performed to evaluate the discriminatory performance of the investigated biomarkers and to estimate threshold values using the Youden index. A two-sided *p*-value < 0.05 was considered statistically significant.

## 3. Results

### 3.1. Patient Characteristics

A total of 122 patients with newly diagnosed MM were included in the study. The median age at diagnosis was 68 years (IQR: 62.2–74.0), and 67 patients (54.9%) were female. Baseline patient characteristics are summarized in Table 1.

According to the ISS stage at diagnosis, 12 patients (9.8%) were classified as stage I, 47 (38.5%) as stage II, and 63 (51.6%) as stage III.

Regarding disease characteristics, the majority of cases were IgG subtype in 72 patients (59.0%), followed by IgA myeloma in 33 (27.0%), and light-chain disease in 17 (13.9%).

Induction therapy consisted of bortezomib, lenalidomide, and dexamethasone (VRd) in 37 patients (30.3%); daratumumab, lenalidomide, and dexamethasone (DRd) in 35 patients (28.7%); bortezomib, cyclophosphamide, and dexamethasone (VCd) in 20 patients (16.4%); daratumumab, bortezomib, lenalidomide, and dexamethasone (Dara-VRd) in 16 patients (13.1%); and daratumumab, bortezomib, melphalan, and prednisone (Dara-VMP) in 14 patients (11.5%).

Autologous stem cell transplantation was performed in 23 patients (18.9%).

During follow-up, 52 patients (42.6%) experienced disease progression or relapse, and 57 patients (46.7%) died. The median follow-up time was 11.3 months (IQR: 4.5–26.6).

### 3.2. Baseline and Post-Induction Changes in Hematologic and Inflammatory Indices

Paired analysis of laboratory parameters obtained at diagnosis and after four cycles of induction therapy revealed significant changes in several hematologic and inflammatory markers.

Hb levels increased significantly after treatment, from a median baseline value of 9.90 g/dL (IQR: 8.10–11.50) to 11.90 g/dL (IQR: 10.60–13.07; *p* < 0.001), suggesting partial hematologic recovery during induction therapy.

Among leukocyte-related parameters, total WBC count decreased significantly (*p* = 0.007), while LYM counts decreased (*p* < 0.001) and MON counts increased modestly (*p* = 0.024). NEU counts did not change significantly (*p* = 0.079). PLT counts and MCV also remained stable (*p* = 0.325 and *p* = 0.627, respectively).

Regarding CBC-derived inflammatory biomarkers, several indices showed significant post-treatment increases, including NLR (*p* = 0.009), PLR (*p* < 0.001), MLR (*p* < 0.001), SII (*p* = 0.022), AISI (*p* = 0.008), SIRI (*p* = 0.004), MCVL (*p* < 0.001), and IIC (*p* < 0.001). RDW also increased significantly after induction therapy (*p* < 0.001).

Overall, induction therapy was associated with substantial dynamic changes in both conventional hematologic parameters and composite inflammatory indices, supporting further evaluation of these indices as potential biomarkers of treatment-related biological response.

The significant dynamic changes observed in hematologic and inflammatory indices following induction therapy are detailed in Table 2.

### 3.3. Association Between Inflammatory Indices and Survival Outcomes

#### 3.3.1. Kaplan–Meier Survival Analysis

Kaplan–Meier survival analysis using median-based stratification did not demonstrate statistically significant differences in PFS or OS for any of the evaluated baseline hematologic or inflammatory biomarkers (Table 3), including MCVL, IIC, NLR, SII, RDW, MCV, PLR, and MLR (all log-rank *p* > 0.05). These findings suggest that simple dichotomization using median cut-off values may not adequately capture the prognostic impact of these biomarkers in this cohort.

**Figure 1:**
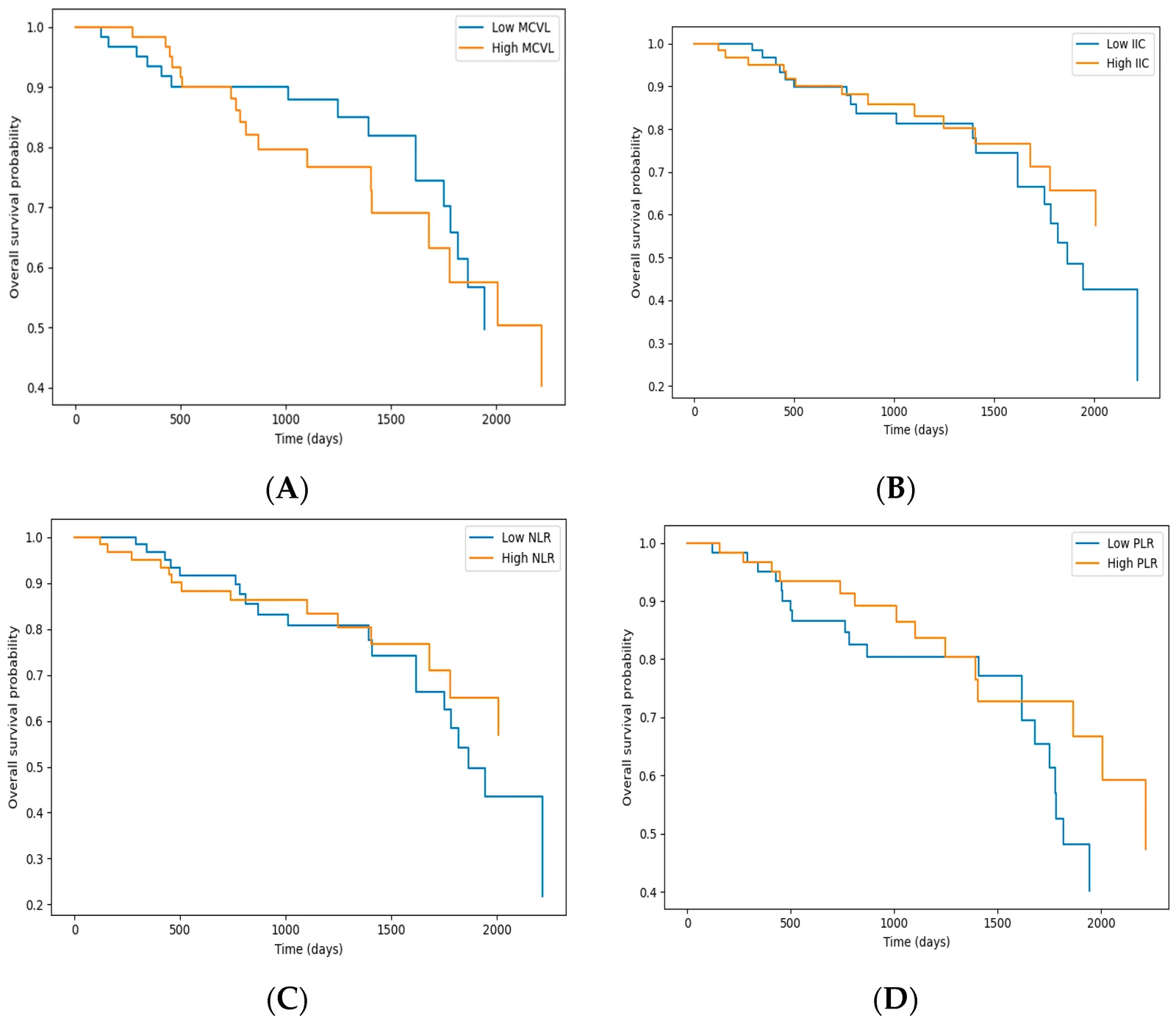
Kaplan–Meier survival curves for overall survival (OS) stratified by median baseline values of (**A**) mean corpuscular volume-to-lymphocyte ratio (MCVL), (**B**) cumulative inflammatory index (IIC), (**C**) neutrophil-to-lymphocyte ratio (NLR), and (**D**) platelet-to-lymphocyte ratio (PLR). No statistically significant differences in survival were observed between high- and low-value groups for any of the evaluated biomarkers.

Kaplan–Meier survival curves for OS stratified by baseline values of NLR, PLR, MCVL, and IIC are presented in Figure 1.

**Figure 2:**
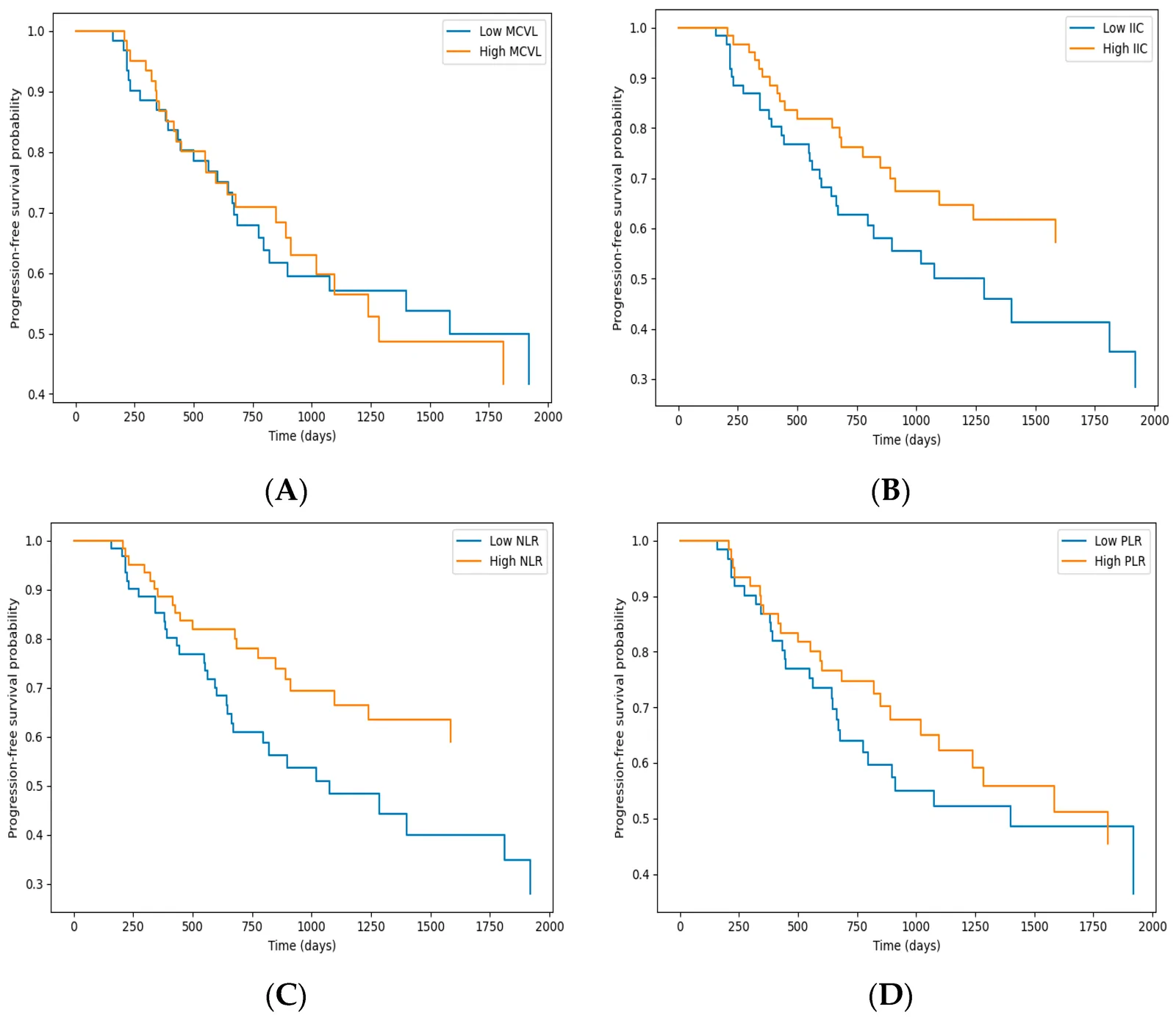
Kaplan–Meier curves for progression-free survival (PFS) stratified by median baseline values of (**A**) mean corpuscular volume-to-lymphocyte ratio (MCVL), (**B**) cumulative inflammatory index (IIC), (**C**) neutrophil-to-lymphocyte ratio (NLR), and (**D**) platelet-to-lymphocyte ratio (PLR). No statistically significant differences in PFS were observed between high- and low-value groups for any of the evaluated biomarkers.

Kaplan–Meier survival curves for PFS, stratified by baseline NLR, PLR, MCVL, and IIC, are presented in Figure 2.

#### 3.3.2. Univariable Cox Regression Analysis

Univariable Cox regression analysis was performed to evaluate the prognostic impact of baseline hematologic and inflammatory indices on PFS and OS, with continuous-variable modeling.

None of the baseline erythrocyte-related parameters (Table 4), including RDW and MCV, were significantly associated with PFS or OS. Similarly, composite erythrocyte-based indices such as MCVL and IIC did not demonstrate significant prognostic value for either survival endpoint (Table 4).

Among leukocyte-derived inflammatory markers, traditional indices, including NLR, PLR, MLR, and SII, were also not significantly associated with PFS or OS when analyzed at baseline.

In contrast, post-induction inflammatory indices (Table 5) demonstrated a stronger association with clinical outcomes. Specifically, higher values of PLR, MLR, AISI, and SIRI measured after four cycles of induction therapy were significantly associated with PFS in univariable analysis. However, no post-induction biomarker, including MCVL and IIC, showed a statistically significant association with OS.

These findings support the hypothesis that dynamic inflammatory changes during treatment may be more informative than baseline measurements for predicting disease progression.

#### 3.3.3. Multivariable Cox Regression Analysis

Multivariable Cox regression analysis was performed to evaluate the independent prognostic value of inflammatory biomarkers after adjustment for clinically relevant variables, including age, disease stage, transplant status, serum creatinine, LDH, and the percentage of bone marrow plasmocytes.

Due to potential collinearity among CBC-derived inflammatory indices, separate multivariable models were constructed for each biomarker.

After adjustment, post-induction PLR, MLR, AISI, and SIRI remained significantly associated with PFS, confirming their independent prognostic value. In contrast, neither MCVL nor IIC demonstrated a statistically significant association with PFS in adjusted models (Table 6).

The proportional hazards assumption was assessed using Schoenfeld residuals. The assumption was not violated in any of the multivariable PFS models, with global test *p*-values ranging from 0.397 to 0.960. In the secondary OS analyses, evidence of non-proportionality was observed for post-induction PLR and MCVL. Because neither marker was significantly associated with OS, these findings did not affect the principal conclusions but indicate that the corresponding OS hazard ratios should be interpreted cautiously.

#### 3.3.4. ROC Analysis for Prediction of PFS and OS (Baseline vs Post-4 Cycles)

ROC analysis was performed to evaluate the predictive performance of inflammatory biomarkers for PFS at both baseline and after four cycles of induction therapy. Cut-off values were determined using the Youden index.

**Figure 3:**
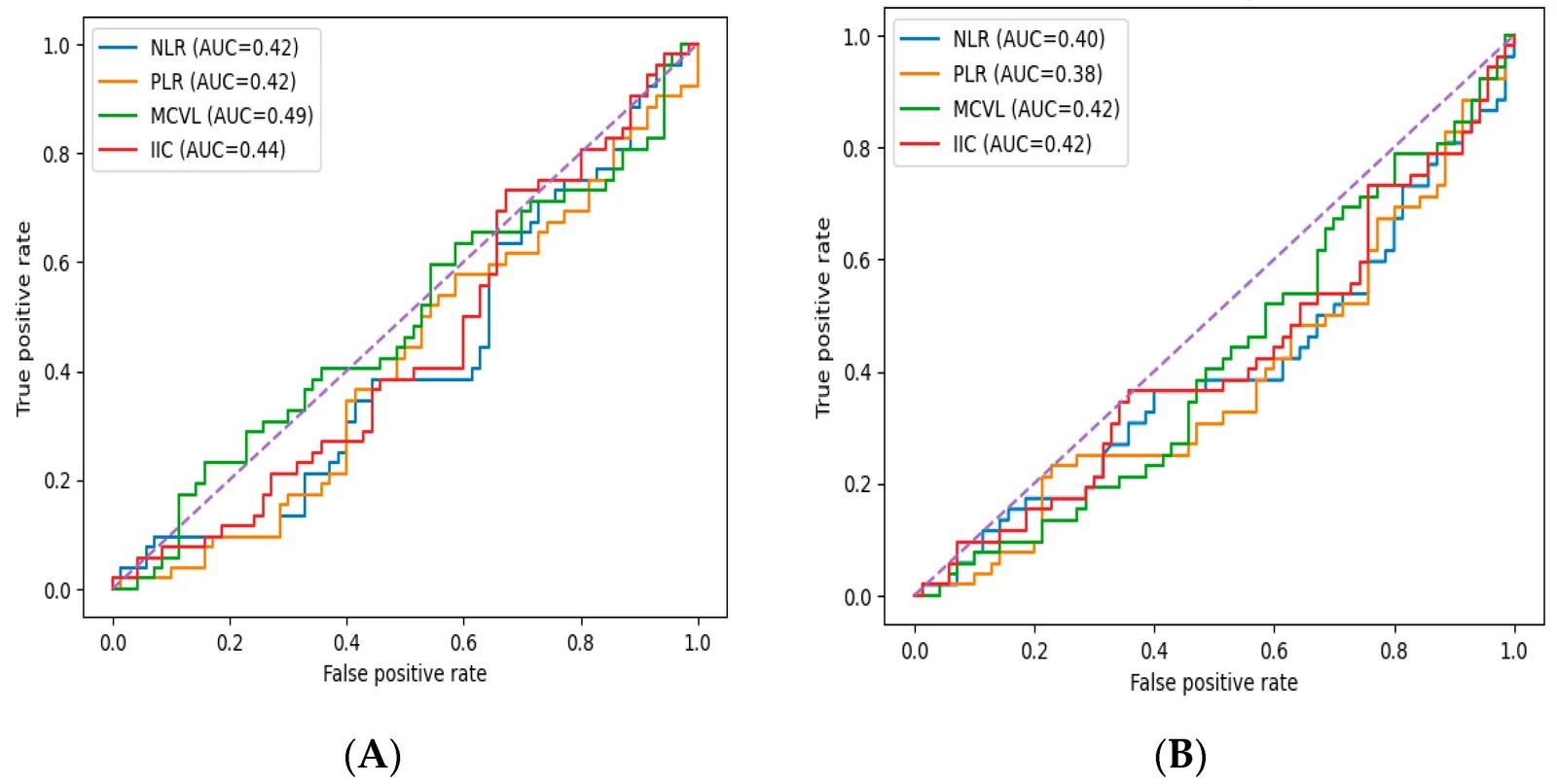
Receiver operating characteristic (ROC) curves illustrating the performance of baseline (**A**) and post-induction (**B**) inflammatory biomarkers in predicting progression-free survival (PFS). The evaluated indices (NLR, PLR, MCVL, and IIC) showed consistently low discriminative capacity, with AUC values below 0.50, indicating poor predictive performance.

At baseline (Table 7, Figure 3), all evaluated biomarkers demonstrated limited discriminative ability, with AUC values < 0.50, including NLR (AUC = 0.42), PLR (AUC = 0.42), MCVL (AUC = 0.49), and IIC (AUC = 0.44).

Similarly, post-induction biomarkers showed poor predictive performance, with AUC values remaining below 0.50 for NLR (AUC = 0.40), PLR (AUC = 0.38), MCVL (AUC = 0.42), and IIC (AUC = 0.42).

These findings indicate that neither baseline nor post-treatment inflammatory indices demonstrated adequate discriminative power to predict PFS in this cohort.

**Figure 4:**
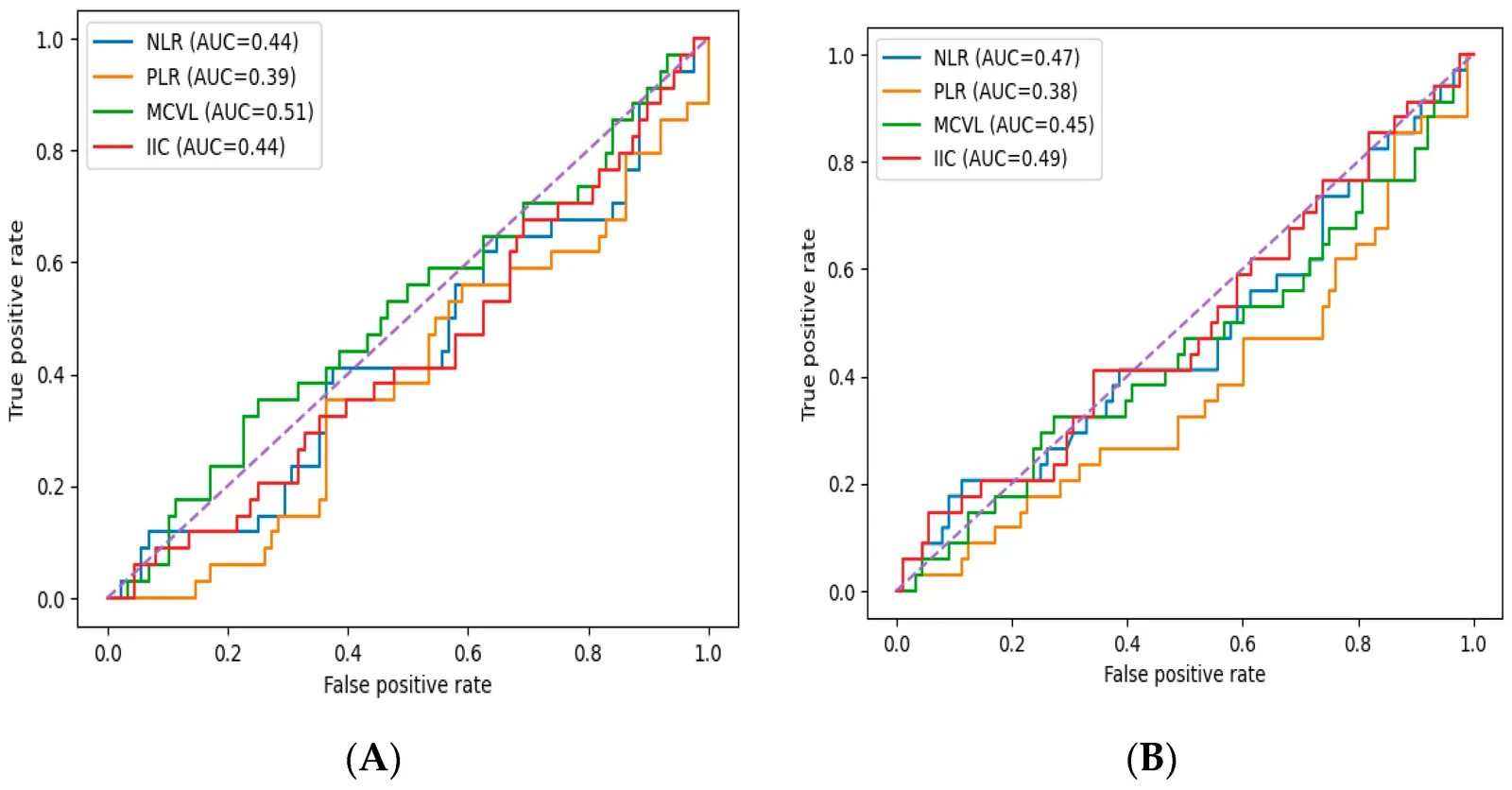
Receiver operating characteristic (ROC) curves illustrating the performance of baseline (**A**) and post-induction (**B**) inflammatory biomarkers in predicting overall survival (OS). The evaluated indices (NLR, PLR, MCVL, and IIC) exhibited consistently low discriminative capacity, with AUC values near 0.50, indicating limited prognostic utility.

ROC curve analysis was performed to assess the predictive performance of inflammatory biomarkers for OS at baseline and after four cycles of induction therapy (Table 8, Figure 4).

At baseline, the discriminative ability of the evaluated biomarkers was limited, with AUC values below 0.50 for NLR (AUC = 0.44), PLR (AUC = 0.39), and IIC (AUC = 0.44). MCVL showed marginal performance (AUC = 0.51), indicating no meaningful predictive capacity.

Similarly, post-induction biomarkers demonstrated poor predictive performance, with AUC values near 0.50 for NLR (AUC = 0.47), PLR (AUC = 0.38), MCVL (AUC = 0.45), and IIC (AUC = 0.49).

Overall, ROC analysis indicated that neither baseline nor post-induction inflammatory indices provided adequate discrimination for OS in this cohort.

## 4. Discussion

In this study, we evaluated the prognostic relevance of multiple hematologic and inflammatory indices in patients with newly diagnosed MM, with particular emphasis on their longitudinal evolution during induction therapy. Two analytical time points were examined: baseline (at diagnosis) and after four cycles of induction treatment, enabling a direct comparison between static and dynamic biomarker assessments. Our findings show that baseline inflammatory indices, including established markers (NLR, PLR, MLR, SII, AISI, SIRI) and the novel erythrocyte-based composite indices MCVL and IIC, were not associated with PFS or OS in this cohort. In contrast, post-induction values of PLR, MLR, AISI, and SIRI were independently associated with PFS in both univariable and multivariable Cox regression analyses, underscoring the potential prognostic relevance of treatment-induced changes in systemic inflammation.

The lack of prognostic significance of baseline NLR, PLR, MLR, and SII in our cohort is consistent with several recent reports highlighting the limited reproducibility of these markers across MM populations. While earlier meta-analyses suggested that elevated NLR is associated with adverse outcomes in MM, subsequent individual studies have produced conflicting results, likely owing to differences in patient selection, treatment regimens, staging systems, and the lack of standardized cut-off values [23,24,25]. In our cohort, Kaplan–Meier analysis using median-based cut-offs confirmed the absence of significant survival differences, and continuous-variable Cox regression models further corroborated these null findings for all baseline indices.

Several biological and methodological factors may explain the limited prognostic value of baseline inflammatory markers in this context. First, the BMME in MM is highly complex, involving bidirectional crosstalk among malignant plasma cells, bone marrow stromal cells, osteoclasts, and immune effector populations [26]. The peripheral blood indices examined here capture only a partial and indirect reflection of this microenvironmental complexity. Second, in newly diagnosed MM, the inflammatory milieu at diagnosis is confounded by a range of disease- and comorbidity-related factors, including infection susceptibility, renal impairment, and anemia-driven erythropoietic stress, all of which can independently alter CBC-derived ratios without necessarily reflecting tumor biology per se [27,28,29]. Third, as demonstrated by the poor discriminative performance of ROC analysis (AUC values at or below 0.50 for all baseline biomarkers), single-timepoint measurements at diagnosis may simply not capture sufficient prognostic signal in this disease setting.

A central and clinically meaningful finding of this study is that post-induction inflammatory indices—specifically PLR, MLR, AISI, and SIRI—were independently associated with PFS after adjustment for age, disease stage, transplant status, serum creatinine, LDH, and bone marrow plasma cell percentage. This observation supports the hypothesis that the inflammatory state after induction therapy more faithfully reflects disease biology and therapeutic response than baseline measurements [30].

Induction therapy in MM—particularly regimens incorporating dexamethasone and alkylating agents—is known to induce peripheral lymphopenia through lymphocyte redistribution and apoptosis, while monocyte counts may be relatively preserved or modestly increased. These treatment-related changes in peripheral blood-cell composition can increase lymphocyte-based inflammatory indices such as NLR, PLR, MLR, AISI, and SIRI. In our cohort, lymphocyte counts decreased significantly (*p* < 0.001), whereas monocyte counts increased modestly (*p* = 0.024), accompanied by significant increases in all five post-induction indices (all *p* < 0.05). The prognostic relevance of these post-induction values may therefore reflect not only treatment-induced immune modulation, but also the extent to which the lymphocyte compartment recovers or remains depleted during induction, a dynamic previously associated with residual disease burden and immune competence in MM [31]. However, these effects may not have been uniform across the cohort because the induction regimens were heterogeneous. Differences in corticosteroid exposure and in the use of proteasome inhibitors, immunomodulatory drugs, alkylating agents, and anti-CD38 monoclonal antibodies may have differentially influenced leukocyte, platelet, and erythrocyte parameters. Accordingly, the observed biomarker dynamics may reflect regimen-specific hematologic and immunomodulatory effects in addition to changes in disease activity.

In addition to regimen-related influences, residual confounding factors may have impacted the biomarker evaluations. While patients with active infections and significant inflammatory disorders were excluded, various treatment- and patient-related variables that could modify peripheral blood cell parameters were not entirely controlled for in this retrospective cohort study. For instance, corticosteroid use can elevate circulating neutrophil levels due to demargination while concurrently decreasing lymphocyte counts through mechanisms such as redistribution and apoptosis, thus affecting NLR, PLR, MLR, AISI, and SIRI [32,33]. Moreover, supportive interventions—such as transfusions, granulocyte colony-stimulating factors, erythropoiesis-stimulating agents, antimicrobial prophylaxis, and other concurrent medications—may significantly influence leukocyte, platelet, and erythrocyte measurements [34,35,36]. Renal impairment may lead to anemia due to reduced erythropoietin production and systemic inflammation. As a result, the post-induction indices should be considered composite measures that represent the interplay of disease burden, treatment exposure, supportive care, and unique patient characteristics, rather than serving as precise indicators of myeloma-related inflammation.

The independent association of post-induction MLR with PFS deserves specific comment. The very low hazard ratio observed for MLR (HR ≈ 0.09) suggests a strong apparent protective effect of higher post-induction MLR values, which is counterintuitive, given that elevated monocyte-to-lymphocyte ratios are generally considered markers of immunosuppression and adverse prognosis [13]. This result should be interpreted with caution. It may reflect a statistical artifact arising from the relatively small sample size, skewed distribution, or residual confounding not fully captured by the multivariable model. Alternatively, it may point to a context-specific biological phenomenon in post-treatment MM, where monocyte-derived myeloid cells may transiently adopt anti-tumoral functions in response to immunomodulatory therapy [37]. This finding warrants prospective validation before any clinical conclusions are drawn.

To our knowledge, this study is among the first systematic evaluations of the MCVL and IIC in patients with MM. The MCVL and the IIC were designed to integrate erythrocyte morphological parameters with leukocyte-derived inflammatory signals, providing a composite measure of both bone marrow dysfunction and systemic immune activation. These indices have demonstrated prognostic value in other malignancies, including colorectal adenocarcinoma, as well as inflammatory conditions such as ulcerative colitis [14,17].

In our cohort, however, neither MCVL nor IIC showed significant associations with PFS or OS at either time point, and ROC analysis confirmed their poor discriminative ability (AUC consistently near or below 0.50). Several explanations may account for this discrepancy with findings in other diseases. In MM, erythrocyte morphological parameters such as RDW and MCV are influenced not only by systemic inflammation but also by disease-specific mechanisms, including bone marrow infiltration by plasma cells, which can cause dyserythropoiesis; treatment-induced myelosuppression; and erythropoietin deficiency secondary to renal impairment—all of which independently alter erythrocyte size and heterogeneity [38]. This multifactorial modulation of erythrocyte indices may dilute their prognostic signal in MM compared with solid tumors or gastrointestinal disease contexts, where erythrocyte–immune coupling may be more specifically informative. Additionally, MCV itself was stable across the treatment period in our cohort (*p* = 0.627), suggesting limited responsiveness of this parameter to induction therapy in MM, thereby constraining the dynamic prognostic capacity of MCVL and IIC.

Although ROC-derived thresholds were estimated using the Youden index, the evaluated biomarkers demonstrated limited discriminatory performance, with AUC values close to or below 0.50. Therefore, these thresholds were considered exploratory and were not used for survival stratification. Instead, Kaplan–Meier analyses were based on median cut-offs to provide balanced descriptive group comparisons, while the primary assessment of prognostic value relied on Cox regression models using continuous biomarker values, thereby avoiding the loss of information associated with dichotomization.

The median follow-up period of 11.3 months must be taken into account when analyzing survival data. While this timeframe facilitates the evaluation of initial progression-related outcomes, it may fall short for a comprehensive assessment of long-term OS, especially in MM, where advancements in treatment have markedly improved survival rates. There is no universally accepted optimal duration for follow-up, as its sufficiency is contingent upon the specific endpoint being analyzed, the frequency and timing of observed events, and the percentage of censored patients. Consequently, any lack of significant associations with OS should be approached with caution and underscores the necessity for validation through extended follow-up.

The present study provides several clinically relevant insights into the use of hematologic and inflammatory biomarkers in MM. First, our findings highlight the limited prognostic value of baseline inflammatory indices, suggesting that reliance on single time-point measurements at diagnosis may be insufficient for accurate risk stratification. Second, and most importantly, our study emphasizes the prognostic relevance of evaluating inflammatory biomarkers after four cycles of induction therapy, an underexplored yet clinically meaningful time point. Post-induction indices, particularly PLR, MLR, AISI, and SIRI, were more closely associated with progression-free survival than their baseline counterparts, supporting the concept that early treatment-induced changes in systemic inflammation may better reflect disease dynamics. Third, this study provides one of the first evaluations of erythrocyte-derived composite indices, such as MCVL and IIC, in MM, suggesting that these markers are limited in their applicability in this disease context.

Taken together, these findings support a shift toward longitudinal biomarker assessment in MM, integrating dynamic inflammatory changes into prognostic evaluation rather than relying solely on baseline parameters. Such an approach may contribute to improved early risk stratification and more personalized treatment strategies.

### Study Limitations

Our study has several limitations. The retrospective design and single-center setting may limit generalizability. Additionally, the lack of detailed cytogenetic and molecular risk stratification parameters, such as R-ISS staging, may have influenced the observed associations. The relatively short median follow-up also restricts the assessment of long-term survival outcomes. However, the study also has notable strengths, including the evaluation of both baseline and longitudinal biomarker dynamics, the inclusion of multiple composite inflammatory indices, and the use of robust statistical methods, including multivariable Cox regression and ROC analysis.

In conclusion, our findings suggest that baseline inflammatory and erythrocyte-derived indices, including the novel composite markers MCVL and IIC, have limited prognostic utility in patients with multiple myeloma. In contrast, post-induction inflammatory markers appear to provide more relevant information regarding disease progression. These results highlight the importance of dynamic biomarker assessment and suggest that future research should integrate longitudinal inflammatory changes with molecular and microenvironmental data to improve risk stratification in MM.

## 5. Conclusions

In this study, we evaluated the prognostic relevance of multiple hematologic and inflammatory indices in patients with newly diagnosed MM, with a particular focus on both baseline values and dynamic changes during induction therapy. Our findings demonstrate that baseline inflammatory biomarkers, including classical indices (NLR, PLR, MLR, SII) and novel composite parameters (MCVL, IIC), have limited prognostic value for PFS and OS. In addition, ROC analysis confirmed the poor discriminative capacity of these markers, highlighting the limitations of static, baseline-based risk stratification. In contrast, post-induction inflammatory indices, particularly PLR, MLR, AISI, and SIRI, showed independent associations with PFS, suggesting that treatment-induced changes in systemic inflammation may better reflect disease dynamics and therapeutic response. Importantly, this study is among the first to investigate erythrocyte-based composite indices, such as MCVL and IIC, in MM. Despite their biological plausibility and reported prognostic relevance in other malignancies, these indices did not demonstrate independent predictive value in our cohort. Overall, our results emphasize that dynamic evaluation of inflammatory biomarkers may provide more clinically relevant prognostic information than baseline measurements alone. Future studies integrating longitudinal inflammatory parameters with molecular and microenvironmental profiling are warranted to improve risk stratification and personalized management in multiple myeloma.

## Figures and Tables

**Table 1:** Baseline Characteristics of the Study Population.

Variable	Value
Patients (*n*)	122
Age, years, median (IQR)	68.0 (62.2–74.0)
Female sex, *n* (%)	67 (54.9)
Myeloma subtype, *n* (%)	
IgG, *n* (%)	72 (59.0)
IgA, *n* (%)	33 (27.0)
Light-chain, *n* (%)	17 (13.9)
Stage at diagnosis, *n* (%)	
Stage I (%)	9.8
Stage II (%)	38.5
Stage III (%)	51.6
Autologous transplant, *n* (%)	23 (18.9)
Progression/Relapse *n* (%)	52 (42.6)
Deaths *n* (%)	57 (46.7)
Follow-up, months, median (IQR)	11.3 (4.5–26.6)

**Table 2:** Comparison of Baseline and Post-4-Cycle Hematologic and Inflammatory Parameters.

Parameter	Baseline Median (IQR)	Post-4 Cycles Median (IQR)	*p*-Value
Hb	9.90 (8.10–11.50)	11.90 (10.60–13.07)	<0.001
WBC	6.00 (4.61–7.70)	5.29 (4.27–6.67)	0.007
NEU	3.59 (2.49–4.95)	3.12 (2.30–4.59)	0.079
LYM	1.65 (1.40–2.21)	1.33 (1.04–1.77)	<0.001
MON	0.41 (0.30–0.58)	0.50 (0.33–0.64)	0.024
PLT	209.00 (172.50–270.00)	230.50 (176.25–272.50)	0.325
RDW	15.50 (13.93–17.30)	16.70 (15.27–17.77)	<0.001
MCV	91.55 (87.12–96.60)	91.80 (87.48–96.97)	0.627
NLR	1.93 (1.40–3.07)	2.33 (1.45–3.50)	0.009
PLR	113.16 (88.79–182.03)	160.23 (116.99–234.98)	<0.001
MLR	0.24 (0.17–0.33)	0.36 (0.25–0.53)	<0.001
SII	392.04 (263.66–778.92)	483.18 (271.47–897.95)	0.022
AISI	188.44 (83.18–347.00)	237.75 (119.78–475.10)	0.008
SIRI	0.89 (0.44–1.36)	1.09 (0.62–2.02)	0.004
MCVL	53.18 (41.14–68.02)	69.11 (50.81–89.91)	<0.001
IIC	2.69 (1.88–4.38)	3.37 (2.17–5.47)	<0.001

**Table 3:** Kaplan–Meier Analysis (Median Cut-off).

Marker	OS *p*-Value	PFS *p*-Value
MCVL	0.674	0.962
IIC	0.211	0.882
NLR	0.143	0.826
SII	0.182	0.843
RDW	0.395	0.854
MCV	0.539	0.389
PLR	0.764	0.507
MLR	0.885	0.639

**Table 4:** Univariable Cox Regression for Baseline Biomarkers.

Marker	PFS		OS	
	HR (95% CI)	*p*-Value	HR (95% CI)	*p*-Value
RDW	1.015 (0.959–1.075)	0.602	1.021 (0.972–1.072)	0.402
MCV	0.997 (0.972–1.023)	0.821	1.006 (0.981–1.032)	0.645
NLR	0.961 (0.837–1.105)	0.578	1.013 (0.919–1.117)	0.795
PLR	0.996 (0.992–1.001)	0.089	0.998 (0.995–1.002)	0.443
MLR	1.370 (0.459–4.093)	0.573	1.063 (0.373–3.030)	0.909
SII	1.000 (0.999–1.000)	0.489	1.000 (1.000–1.000)	0.832
AISI	1.000 (1.000–1.000)	0.271	1.000 (1.000–1.000)	0.719
SIRI	1.030 (0.978–1.086)	0.264	0.990 (0.912–1.074)	0.803
MCVL	1.000 (0.991–1.009)	0.932	1.003 (0.994–1.013)	0.473
IIC	0.981 (0.900–1.068)	0.657	1.000 (0.933–1.071)	0.990

**Table 5:** Univariable Cox Regression for Post-Induction Biomarkers.

Marker	PFS		OS	
	HR (95% CI)	*p*-Value	HR (95% CI)	*p*-Value
RDW	1.045 (0.990–1.103)	0.112	1.018 (0.970–1.068)	0.466
MCV	1.002 (0.977–1.028)	0.879	1.004 (0.980–1.029)	0.739
NLR	0.909 (0.801–1.031)	0.136	1.017 (0.944–1.096)	0.660
PLR	0.996 (0.993–0.999)	0.016	1.000 (0.997–1.002)	0.879
MLR	0.094 (0.020–0.441)	0.003	1.645 (0.584–4.634)	0.346
SII	0.999 (0.999–1.000)	0.063	1.000 (1.000–1.000)	0.853
AISI	0.998 (0.997–1.000)	0.007	1.000 (0.999–1.000)	0.732
SIRI	0.740 (0.575–0.954)	0.020	1.003 (0.873–1.153)	0.962
MCVL	0.994 (0.986–1.002)	0.123	0.999 (0.993–1.006)	0.825
IIC	0.953 (0.884–1.026)	0.201	1.012 (0.968–1.058)	0.596

**Table 6:** Multivariable Cox Regression Analysis for Progression-Free Survival (PFS) and Overall Survival (OS).

Marker	PFS		OS	
	Adjusted HR (95% CI)	*p*-Value	Adjusted HR (95% CI)	*p*-Value
PLR	0.996 (0.993–0.999)	0.018	1.000 (0.997–1.002)	0.879
MLR	0.090 (0.019–0.431)	0.003	1.645 (0.584–4.634)	0.346
AISI	0.998 (0.997–1.000)	0.007	1.000 (0.999–1.000)	0.732
SIRI	0.739 (0.572–0.955)	0.021	1.003 (0.873–1.153)	0.962
MCVL	0.994 (0.986–1.002)	0.133	0.999 (0.993–1.006)	0.825
IIC	0.954 (0.885–1.028)	0.213	1.012 (0.968–1.058)	0.596

Multivariable Cox regression models adjusted for age, stage, transplant, creatinine, LDH, and plasma cell percentage.

**Table 7:** ROC Analysis for Prediction of PFS (Baseline vs. Post-4 Cycles).

Marker	Timepoint	Cut-Off	AUC
NLR	Baseline	0.73	0.42
PLR	Baseline	478.75	0.42
MCVL	Baseline	74.55	0.49
IIC	Baseline	2.05	0.44
NLR	Post-induction	14.33	0.40
PLR	Post-induction	35.38	0.38
MCVL	Post-induction	22.64	0.42
IIC	Post-induction	11.04	0.42

**Table 8:** ROC Analysis for Prediction of OS (Baseline vs. Post-4 Cycles).

Marker	Timepoint	Cut-Off	AUC
NLR	Baseline	5.71	0.44
PLR	Baseline	—	0.39
MCVL	Baseline	67.69	0.51
IIC	Baseline	0.83	0.44
NLR	Post-induction	5.75	0.47
PLR	Post-induction	35.38	0.38
MCVL	Post-induction	86.82	0.45
IIC	Post-induction	11.04	0.49

## Data Availability

The raw data supporting the conclusions of this article will be made available by the authors on request.
